# Validity of actigraphs uniaxial and triaxial accelerometers for assessment of physical activity in adults in laboratory conditions

**DOI:** 10.1186/1756-6649-13-5

**Published:** 2013-11-26

**Authors:** Louise A Kelly, Duncan GE McMillan, Alexandra Anderson, Morgan Fippinger, Gunnar Fillerup, Jane Rider

**Affiliations:** 1 Department of Exercise Science, California Lutheran University, 60 W. Olsen Road, #3400, Thousand Oaks, CA 91360, USA; 2 Packet Photonics Inc, Santa Barbara, CA, USA

**Keywords:** Actigraph, Triaxial, Accelerometers, Young adults, Validation

## Abstract

**Background:**

Few studies to date have directly compared the Actigraphs GT1M and the GT3X, it would be of tremendous value to know if these accelerometers give similar information about intensities of PA. Knowing if output is similar would have implications for cross-examination of studies. The purpose of the study was to assess the validity of the GT1M and the GT3X Actigraph accelerometers for the assessment of physical activity against oxygen consumption in laboratory conditions.

**Methods:**

Forty-two college-aged participants aged 18-25 years wore the GT1M and the GT3X on their right hip during treadmill exercise at three different speeds, slow walking 4.8 km^.^h^-1^, fast walking 6.4 km^.^h^-1^, and running 9.7 km^.^h^-1^). Oxygen consumption was measured minute-by minute using a metabolic system. Bland-Altman plots were used to assess agreement between activity counts from the GT3X and GT1M, and correlations were assessed the ability of the accelerometers to assess physical activity.

**Results:**

Bias for 4.8 km^.^h^-1^ was 2814.4 cpm (limits 1211.3 to 4417.4), for 6.4 km^.^h^-1^ was 3713.6 cpm (limits 1573.2 to 5854.0), and for 9.7 km^.^h^-1^ was−3811.2 cpm (limits 842.1 to 6780.3). Correlations between counts per minute for the GT1M and the GT3X were significantly correlated with VO_2_ (r = 0.881, p < 0.001; r = 0.810, p < 0.001 respectively).

**Conclusion:**

The present study showed that both the GT1M and the GT3X accurately measure physical activity when compared to oxygen consumption.

## Background

Over the past two decades, the use of accelerometry has become an increasingly popular. As a method for the assessment of physical activity (PA) they provide good evidence of validity and reliability [1,2]. The increase in accelerometry use may be a result for the need of accurate and objective techniques. As a result, a number of accelerometers have become commercially available. One of the most widely used accelerometers in PA research is the Actigraph’s Uniaxial GT1M (Actigraph, Pensacola, FL) and more recently the company released the triaxial GT3X (Actigraph, Pensacola, FL) [3]. These accelerometers are based on differential capacitance sensor using the Microelectromechanical Systems (MEMS) lithography technology to manufacture the tiny measurement devices [4-6]. These MEMS based accelerometers are designed to have an excellent force range and bandwidth of operation suited to measuring physical activity from low frequency (>0.1 Hz) right up to the kHz range. In addition their resonant frequency is far above the target frequency range of interest and will not distort the measurements of human activity. Differential capacitance may also be defined as “parallel plate capacitance” after the typical design of the MEMS capacitor sensor Capacitors can be thought of as electrical storage devices that are constantly discharging. Differential capacitance is a parameter used to characterize electrical double layers i.e. the two electrical parallel plates in accelerometers [4-6].

The sensor works by having one plate fixed and the other plate mounted in such a way that it moves when it experiences an external force on the device. The movement of the plate relative to the other fixed plate generates a change in the stored charge (Surface charge) and the voltage stored between the two plates (Electric surface potential), it is the rate of change of stored charge divided by the rate of change of the voltage that defines the differential capacitance. While the GT1M contains an ADXL320 acceleration sensor, which, measures both static and dynamic accelerations, the GT3X contains an ADXL335 accelerometer (Analog Devices). Although the accelerometer is different in the GT3X, the structure and theory of operations of the ADXL335 is similar to that of the ADXL320 [4-6]. In addition, the GT3X provides inclinometer output and uses vector magnitude data form three axes. The GT3X measures acceleroations in three individual plans of motion, the vertical (VT), antero-posterior (AP), and medio-lateral (ML) and provides activity counts as a composite vector magnitude of these three axes (VM3). So in theory, the GT3X should provide a more accurate assessment of physical activity.

While increasing number of adult and pediatric studies use Actigraphs uniaxial accelerometers [7-15], and various versions have been introduced over the years, numerous investigators have reported on the validity to assess physical activity in the laboratory and in field settings [16-19], to estimate energy expenditure [15,20-25] and in the comparison to other activity monitors from other manufacturers [26-29]. However, few studies to date have directly compared the Actigraphs GT1M and the GT3X, it would be of tremendous value to know if these accelerometers give similar information about intensities of PA. Knowing if output is similar would have implications for cross-examination of studies. Therefore, the purpose of the study was to assess the validity of the GT1M and the GT3X Actigraph accelerometers for the assessment of physical activity against oxygen consumption.

## Methods

### Participants

Data was analyzed from 42 healthy, recreationally active, adults (23 males, 19 females) recruited from the California Lutheran University student, staff and faculty population along with surrounding communities through word of mouth. Participants were required to meet the following study entry inclusion criteria: 1) age 18-30 years, and 2) BMI 20-24 according to the WHO international classifications of adult BMI [30]. Part of our protocol included running at a modest pace, therefore, participants were excluded if they were taking medications known to affect oxygen consumption, physical activity, body composition, diagnosed with any syndromes known to affect oxygen consumption, physical activity, body composition or self reported smokers. Written informed consent was obtained from participants before testing began. The Institutional Review Board of the California Lutheran University approved this study, and all procedures were performed in accordance with standards outlined in the Helsinki Declaration.

### Study design

Using a similar methodology as described by Freedson et al. [13], each accelerometer was initialized per manufacturers instructions, and the sampling period was set at 1 minute, raw output was expressed as counts per minute (cpm) prior to each testing session. To evaluate the validity of the GT1M (Actigraph, Pensacola, FL) and the GT3X (Actigraph, Pensacola, FL) against oxygen consumption, all participants wore both accelerometers simultaneously, over the right hip and secured it with the same adjustable elastic belt and buckle supplied by the manufacturers. After a standardized 10 minute familiarization period on a calibrated treadmill, participants performed 6 minutes of the following exercise conditions: slow walking (4.8 km^.^h^-1^), fast walking (6.4 km^.^h^-1^), and running (9.7 km^.^h^-1^), and the order of exercise conditions was random across participants. All exercise bouts were performed at 0% grade due to the known limitations of the GT1M [15]. Each exercise bout was separated by a 5-minute rest period, this rest period was standardized for each participant, as previously described by Freedson et al. [13]. While study participants reported no know contradictions to exercise, as a precaution, exercise heart rate was monitored using a Polar T31 transmitter and receiver (Lake Success, NY, US). Following the testing session both accelerometers were immediately downloaded as per the manufactures instructions using firmware version 5.10.

### GT1M actigraph

The Actigraph GT1M Activity monitor is a small (3.8 × 3.7 × 1.8 cm) light (27 g) uniaxial accelerometer, which is housed in a plastic case. The GT1M is a solid-state micro-electro-mechanical systems (MEMS). It is designed to measure and record time varying accelerations ranging in magnitude from 0.05 to 2Gs approximately with a frequency response of 0.25 to 2.50 HZ. These parameters were chosen as they best detect normal human motion and reject motion from other sources [4-6]. The GT1M measures acceleration in Vertical (VT) plane only [4-6,14].

The GT1M accelerometer now uses a highly accurate solid-state accelerometer, which undergoes a precise batch manufacturing process to ensure high repeatability. Esigler et al. [31] reported the overall intra and inter-instrumentation reliability of the GT1M for counts was 2.9 and 3.5% respectively and for steps 1.1 and 1.2% respectively. The filter is now implemented within the software of the device, thus removing unit-to-unit variability due to this source, leaving only the accelerometer vendor’s initial tolerance specification on sensitivity as the primary source of error. The accelerometer vendor claims the devices are manufactured to ensure that the initial tolerance specification on sensitivity only varies by ± 10% [4-6].

### GT3X actigraph

The GT3X is lightweight and compact with a weight of 27 grams and dimensions of (3.8 cm × 3.7 cm × 1.8 cm). The GT3X activity monitor uses a solid-state triaxial accelerometer to collect motion data on three axes for the highest levels of analytic capabilities available.

ActiGraph GT3X activity monitor accurately and consistently measures and records time varying accelerations ranging in magnitude from approximately 0.05 to 2.5 G’s. The accelerometer output is digitized by a twelve-bit (12) Analog to Digital Convertor (ADC) at a rate of thirty times per second (30 Hertz). Once digitized, the signal passes through a digital filter that band-limits the accelerometer to the frequency range of 0.25 to 2.5 Hz. This frequency range has been carefully chosen to detect normal human motion and to reject changing accelerations within the pass band. The GT3X has also demonstrated high reliability with an intra-class correlation coefficient for activity counts of 0.97 [32]. The GT3X measures acceleration in three individual orthogonal planes (VT, AP, and medio-lateral (ML)) and provides activity counts as a composite vector magnitude of these three axes (VM3) [4-6,14].

### Indirect calorimetry

Expired gases were collected in a mixing chamber with samples taken every 15 seconds; 30-second averages were analyzed via a Parvo-Medics: True One 2400 Metabolic System (Sandy, Utah, US). Analyzers were calibrated before and after each testing session using verified calibration gasses. Steady state energy expenditure (kcal^.^min-1) was calculated by averaging the final 3 minutes of each exercise condition.

### Anthropometric measurements

Participants were measured in light indoor clothing without shoes. Height and weight were measured in triplicate using a beam medical scale and wall-mounted stadiometer, to the nearest 0.1 kg and 0.1 cm, respectively. The mean of the measurements were used to calculate body mass index (BMI) (kg/m^2^).

### Statistical analysis

Prior to statistical analysis, each of the three methods was tested for normality using the Shapiro-Wilk test. Pearson bivariate correlations were used to assess the simple relationship between VO_2_ and activity counts from the GT1M and the GT3X. Bland-Altman [33] plots were used to assess agreement between activity counts from the GT3X and GT1M. For all analyses, statistical significance was set at an alpha level of 0.05.

## Results

**Figure 1:**
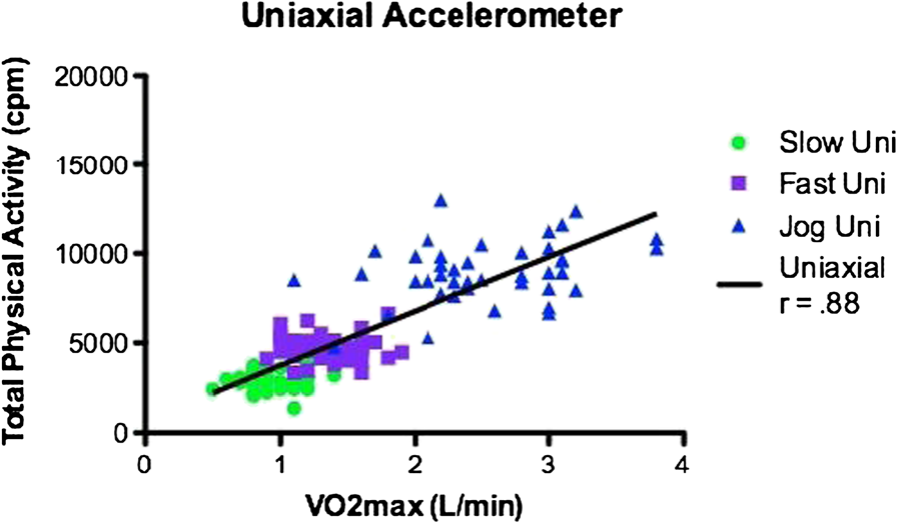
Correlation of GT1M and VO2(L/min).

**Figure 2:**
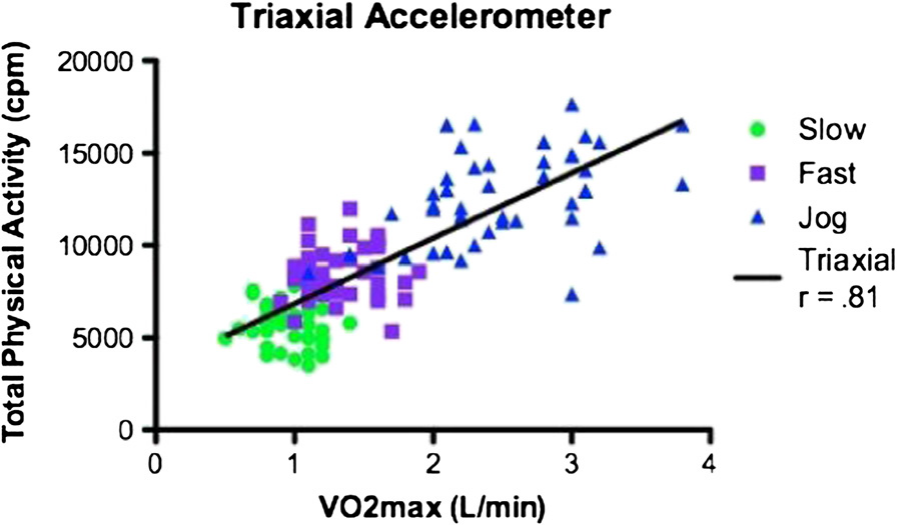
Correlation of GT3X and VO2 max (L/min).

**Figure 3:**
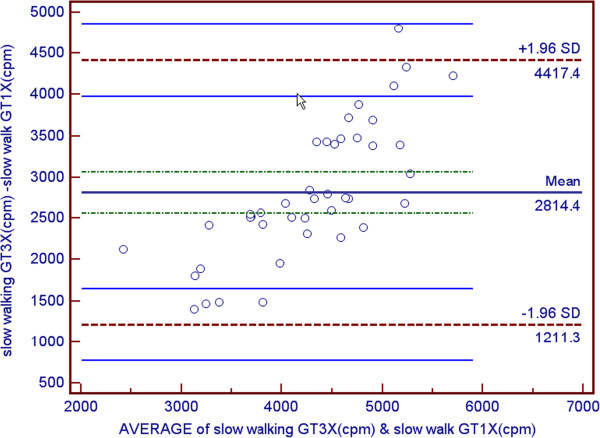
Bland/Altman plot for GT1M and GT3X at slow walking.

**Figure 4:**
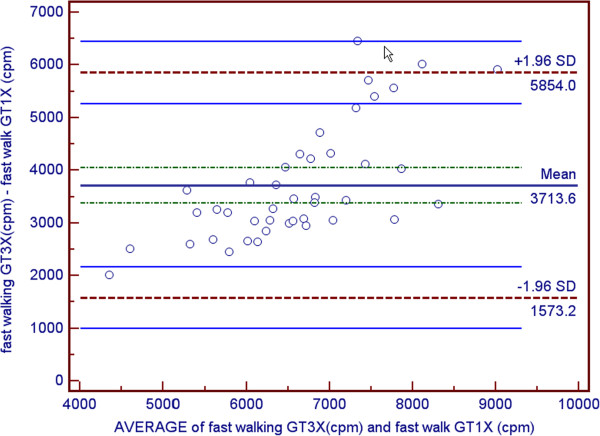
Bland/Altman plot for GT1M and GT3X at fast walking.

**Figure 5:**
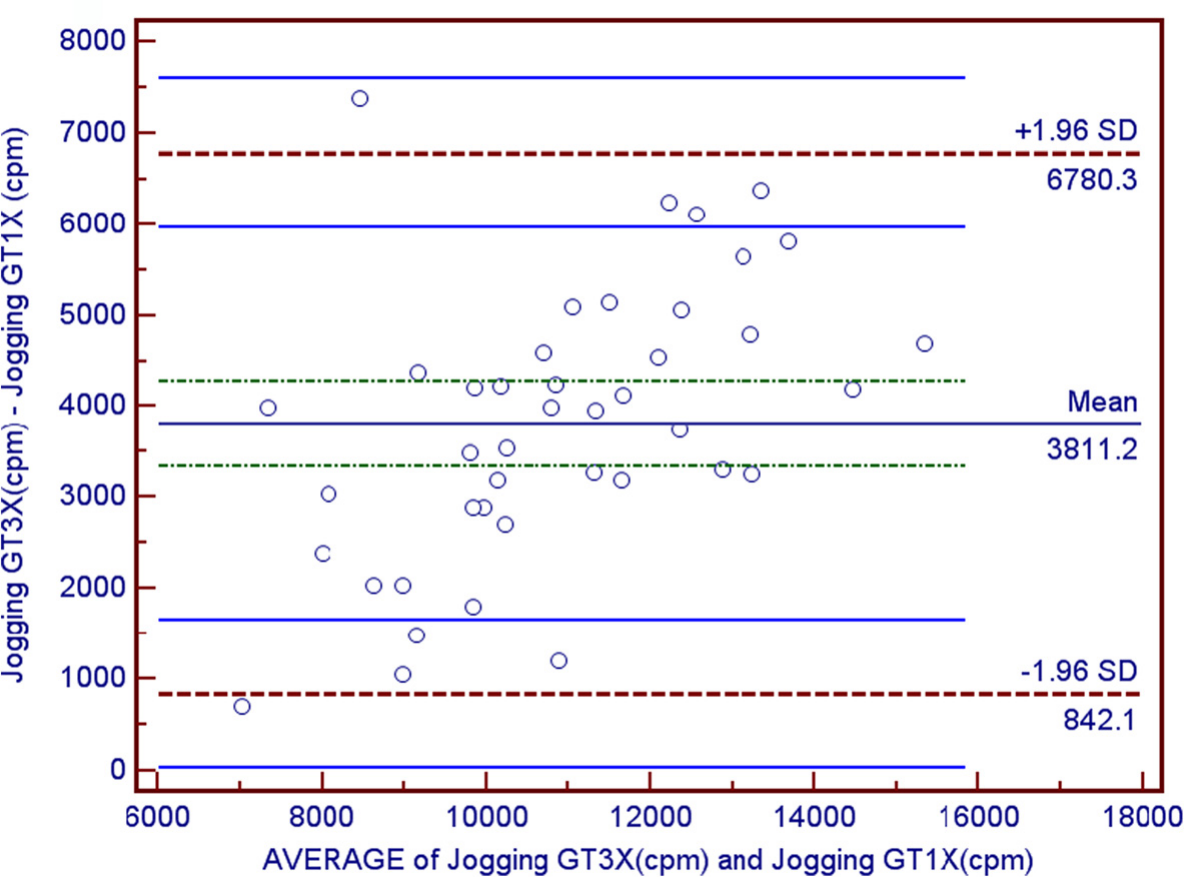
Bland/Altman plot for GT1M and GT3X at Running.

Participants characteristics and means and ± SD for VO2 and accelerometer data are shown are shown in Table 1 and Table 2 respectively. The average age was 21.57 ± 2.73 years. Since no gender differences were observed, all data analysis was performed on the total sample. As a result of attrition due to restriction of range, correlations were performed on the total sample instead of individual speeds. Correlations between counts per minute for the GT1M and the GT3X were strongly positively and statistically significantly correlated (r = 0.937, p < 0.001) (see Figures 1 and 2). Both the GT1M and the GT3X accelerometers count/minute were significantly positively correlated with VO_2_ (r 0.881, p < 0.001; r = 0.810, p < 0.001 respectively). Bias for 4.8 km^.^h^-1^ was 2814.4 cpm (limits 1211.3 to 4417.4), for 6.4 km^.^h^-1^ was 3713.6 cpm (limits 1573.2 to 5854.0), and for 9.7 km^.^h^-1^ was−3811.2 cpm (limits 842.1 to 6780.3) (see Figures 3, 4 and 5).

**Table 1:** Participants characteristics (mean ± SD)

**Characteristics**	**Group (n = 42)**
Age (yrs)	21.57 ± 2.73
Height (M)	1.74 ± 0.10
Weight (kg)	76.94 ± 15.35
BMI (kg/m2)	25.26 ± 3.25

note: all data are means and stand deviations unless otherwise stated.

**Table 2:** Mean (± SD) for VO2 and activity county for GT1M and GT3X

**Speed**	**VO**^**2**^	**GT1M**	**GT3X**
**(km**^**.**^**h**^**-1**^**)**	**(l/min)**	**(cpm)**	**(cpm)**
4.8	0.93 ± 0.2	2874.45 ± 479.9	5688.83 ± 1072.3
6.4	1.32 ± 0.3	4756.52 ± 707.4	8470.14 ± 1402.9
9.7	2.54 ± 0.5	8962.89 ± 1686.8	12774.09 ± 2413.8

note: all data are means and stand deviations unless otherwise stated.

## Discussion

The GT3X and other triaxial accelerometers were developed under the assumption that more planes are better [34,35]. By measuring motion in more than one plane, these monitors might be better able to quantify physical activity more effectively than uniaxial accelerometers. Indeed, several authors have suggested specifically that triaxial accelerometers may be more sensitive than uniaxial accelerometers to the torsional, non-vertical movement associated with every day movement [36-39]. The present study aimed to assess the ability of two accelerometers to measure physical activity compared to the criterion oxygen consumption. It was assumed that the GT3X accelerometer would assess PA levels more accurately than the GT1M as this triaxial accelerometer measures movements in the 3 dimensions of space, whereas the uniaxial accelerometer measures movement in one dimension only [40,41]. While results from other studies showed that triaxial accelrometers measured physical activity and energy expenditure in adults and children more accurately than uniaxial accelerometers [42,43], our results show that the additional information obtained from 3 dimensional movements is not more efficient or more accurate than the uniaxial accelerometer when compared to oxygen consumption.

There may be a number of reasons the GT3X was not more accurate than the GT1M, firstly, it is possible that walking and running on a treadmill did not illicit enough of a force to register accurately on any other axis other than the vertical. Furthermore, walking, fast walking and running may not have produced enough deviation of sway, or had a lower impact on anteroposterior and mediolateral axis. In fact, there may be evidence to suggest that the vertical is indeed the most important axis [42]. Secondly, as walking, fast walking and running were conducted on a treadmill with no incline there was very little opportunity for anteroposterior and mediolateral axis movements.

Sasaki et al. [14], study using an older sample population and a much firmware found similar results to our study. The authors concluded that VT counts from the GT1M and the GT3X were analogous and therefore if data have been collected in the VT plan only then data can be directly comparable. The results of our study are also similar to those of Welk et al. [43], who predicted energy expenditure using three different accelerometers the ActiGraph (uniaxial), Tritrac-RT3 (triaxial), and BioTrainer (bidirectional). While energy expenditure estimates increased in all three activity monitors during running, walking and lawn mowing, all three monitors significantly underestimated the energy cost of more static and/or complex movement patterns by approximately 50%. The Welk et al. [43], study concluded that the assessment of multiple dimensions of movement do not provide enough extra information to fill the considerable “energy gap” experienced by these monitors. Another study by Hänggi et al. [44], investigated the comparability of the GT3X to the GT1M in children during a semi structured activity laboratory setting concluded that the monitors differ for certain activities, and posture classification by the GT3X should be interpreted with care, as misclassifications are common.

However, results from an older study by Eston et al. [37] contradict our findings. The Eston study compared the accuracy of heart rate monitoring, pedometry, triaxial accelerometry, and uniaxial accelerometry for estimating oxygen consumption during typical children’s activities. While all measurements significantly correlated with VO_2_, the authors concluded that triaxial accelerometers provide more accurate assessments of physical activity. It is worth noting that both accelerometers used in this study were old versions of the WAM 7146 uniaxial and the Tritrac R-3-D triaxial accelerometer. Similarly, a study by Vanhelst et al. [45], using a different triaxial accelerometer the RT3, compared equivalence and agreement of physical activity output data collected by a Research Tri-axial accelerometer (R3T) during walking and running on a treadmill versus on land in fifty healthy participants. The results showed good agreement between the counts obtained on the treadmill and on land (P < 0.05). The study concluded that the RT3 may be used in a laboratory and extrapolated to data obtained on land.

While a major strength of this study was the comparison of the uniaxial and triaxial accelerometers to the gold standard technique of oxygen consumption, a number of study limitations are worth noting. Firstly, this was a lab-based study and therefore we cannot infer that results from the current study would reflect free living conditions. Secondly, we only had 3 speeds, slow walking (4.8 km^.^h^-1^), fast walking (6.4 km^.^h^-1^), and running (9.7 km^.^h^-1^). It could be argued that we may have been more accurate at higher speeds, however, Sasaki et al. [14], showed that at higher speeds of 9.7 and 12 kmh-1 activity counts remained relatively stable. We chose not to increase our speed as to avoid this plateau or “ceiling” effect, which is a well know limitation of accelerometers [15,46]. Thirdly, our recovery period between each bout of exercise was 5 minutes and this standardized for each participant. This recovery period may not have been a sufficient amount of time for some individuals. However, as our participants were young and physically fit, we believe this period to be adequate. Fourthly, current research has demonstrated that low frequency extension (LFE) increases sensitivity for low intensity activities [3]. As the current study was lab based and did not involve low intensity activities such as sitting, watching television the LFE was not activated. Finally, our sample size was relatively small and very homogenous, so our results may not be generalizable.

## Conclusion

In conclusion, the present study showed that using a newer firmware that both the GT1M and the GT3X accurately measure PA when compared to oxygen consumption, and therefore if data has been collected using the VT axis then a comparison among studies is directly comparable. Future studies should look at assessing validity of both accelerometers in a more diverse physical activity’s, under lifestyle setting and with multiple age ranges.

## Consent

All participants completed an informed consent in person.

## Competing interests

The authors declare that they have no competing interests.

## Authors’ contributions

Dr. Kelly had full access to all of the data in the study and takes responsibility for the integrity of the data and the accuracy of the data analyses. Study concept and design: LK, DMCM, JR. Acquisition of data: LK, DMCM, AA, GF, MF. Analysis and interpretation of data: LK, DMCM. Drafting of the manuscript: LK, DMCM, AA, JR. Critical revision of the manuscript for important intellectual content: LK, DMCM, JR, AA, GF, MF. Statistical analysis: LK. Obtaining funding: LK. Administrative, technical, or material support: AA, GF, MF. Study supervision: LK, DMCM, JR. All authors read and approved the final manuscript.

## Pre-publication history

The pre-publication history for this paper can be accessed here:

http://www.biomedcentral.com/1756-6649/13/5/prepub

## Support

This work was supported by Swenson Summer Research Fellowship.

## References

[B1] Eyler AA Brownson RC Bacak SJ Housemann RA The epidemiology of walking for physical activity in the United States Med Sci Sports Exerc 2003 35 9 1529 1536 10.1249/01.MSS.0000084622.39122.0C 12972873

[B2] Fitzhugh EC Thompson DL Leisure-time walking and compliance with ACSM/AHA aerobic-related physical activity recommendations: 1999-2004 NHANES J Phys Act Health 2009 6 4 393 402 19842452 10.1123/jpah.6.4.393

[B3] Cain KL Conway TL Adams MA Husak LE Sallis JF Comparison of older and newer generations of ActiGraph accelerometers with the normal filter and the low frequency extension Int J Behav Nutr Phys Act 2013 25 10 51 10.1186/1479-5868-10-51 PMC3641979 23618461

[B4] Actigraph Actilife users manual, 2008 2008 Retrieved from http://www.theactigraph.com/index.php?option=com_docman&task=cat_view&gid=53&Itemid=64

[B5] Actigraph GT1M specifications, 2008 2008 Retrieved from http://theactigraph.com/index.php?option=com_docman&task=cat_view&gid=70&Itemid=64

[B6] Actigraph GT1M specifications Retrieved from http://www.theactigraph.com/products/gt3x/

[B7] Jackson DM Reilly JJ Kelly LA Montgomery C Grant S Paton JY Objectively measured physical activity in a representative sample of 3-to 4-year old children Obes Res 2003 11 420 425 10.1038/oby.2003.57 12634440

[B8] Reilly JJ Jackson DM Montgomery C Kelly LA Slater C Grant S Paton JY Levels of total energy expenditure and physical activity in modern children Lancet 2004 363 211 212 10.1016/S0140-6736(03)15331-7 14738795

[B9] Kelly LA Reilly JJ Grant S Paton JY Low physical activity levels and high levels of sedentary behavior are characteristic of rural Irish primary school children Ir Med J 2005 98 138 141 16010780

[B10] Kelly LA Reilly JJ Fisher A Montgomery C Williamson A McColl JH Paton JY Grant S Effect of socio-economic status on objectively measured physical activity Arch Dis Child 2006 9 35 38 16239246 10.1136/adc.2005.080275 PMC2083107

[B11] Reilly JJ Kelly LA Montgomery C Williamson A Fisher A McColl JH Lo Conte R Paton JY Grant S Movement and activity glasgow intervention in children (MAGIC): cluster randomised controlled trial for the prevention of obesity BMJ 2006 333 7577 1041 1043 10.1136/bmj.38979.623773.55 17028105 PMC1647320

[B12] Kelly LA Reilly JJ Jackson DM Montgomery C Grant S Paton JY Tracking of physical activity and sedentary behavior in young children Pediatr Exerc Sci 2007 19 51 60 17554157 10.1123/pes.19.1.51

[B13] Freedson PS Melanson E Sirard J Calibration of the computer science and applications: Inc. accelerometer Med Sci Sports Exerc 1998 30 5 777 781 10.1097/00005768-199805000-00021 9588623

[B14] Sasaki JE Dinesh J Freedson PS Validation and comparison of ActiGraph activity monitors J Sci Med Sport 2011 14 411 416 10.1016/j.jsams.2011.04.003 21616714

[B15] Brage S Wedderkopp N Franks PW Anderson LB Froberg K Re-examination of validity and reliability of the CSA monitor in walking and running Med Sci Sports and Exerc 2003 35 1447 1454 10.1249/01.MSS.0000079078.62035.EC 12900703

[B16] Janz KF Validation of the CSA accelerometer for assessing children’s physical activity Med Sci Sports Exerc 1994 26 3 369 375 8183103

[B17] Melanson EL Jr Freedson PS Validity of the computer science and applications, Inc. (CSA) activity monitor Med Sci Sports Exerc 1995 27 6 934 940 7658958

[B18] Bassett DR Jr Validity and reliability issues in objective monitoring of physical activity Res Q Exerc Sport 2000 71 2 Suppl S30 S36 10925822

[B19] Hendelman D Miller K Baggett C Debold E Freedson P Validity of accelerometry for the assessment of moderate intensity physical activity in the field Med Sci Sports Exerc 2000 32 9 Suppl S442 S449 10993413 10.1097/00005768-200009001-00002

[B20] Swartz AM Strath SJ Bassett DR Jr O’Brien WL King GA Ainsworth BE Estimation of energy expenditure using CSA accelerometers at hip and wrist sites Med Sci Sports Exerc 2000 32 9 Suppl S450 S456 10993414 10.1097/00005768-200009001-00003

[B21] King GA Torres N Potter C Brooks TJ Coleman KJ Comparison of activity monitors to estimate energy cost of treadmill exercise Med Sci Sports Exerc 2004 36 7 1244 1251 10.1249/01.MSS.0000132379.09364.F8 15235333

[B22] Crouter SE Churilla JR Bassett DR Jr Estimating energy expenditure using accelerometers Eur J Appl Physiol 2006 98 6 601 612 10.1007/s00421-006-0307-5 17058102

[B23] Crouter SE Clowers KG Bassett DR Jr A novel method for using accelerometer data to predict energy expenditure J Appl Physiol 2006 100 4 1324 1331 16322367 10.1152/japplphysiol.00818.2005

[B24] Rothney MP Brychta RJ Meade NN Chen KY Buchowski MS Validation of the ActiGraph two-regression model for predicting energy expenditure Med Sci Sports Exerc 2010 42 9 1785 1792 10.1249/MSS.0b013e3181d5a984 20142778 PMC2919650

[B25] Crouter S Kuffel E Haas JD Frongillo EA Bassett DR Jr Refined two-regression model for the ActiGraph accelerometer Med Sci Sports Exerc 2010 42 5 1029 1037 10.1249/MSS.0b013e3181c37458 20400882 PMC2891855

[B26] Tudor-Locke C Ainsworth BE Thompson RW Matthews CE Comparison of pedometer and accelerometer measures of freeliving physical activity Med Sci Sports Exerc 2002 34 12 2045 2051 10.1097/00005768-200212000-00027 12471314

[B27] Le Masurier G Tudor-Locke C Comparison of pedometer and accelerometer accuracy under controlled conditions Med Sci Sports Exerc 2003 35 5 867 871 10.1249/01.MSS.0000064996.63632.10 12750599

[B28] Rothney MP Apker GA Song Y Chen KY Comparing the performance of three generations of ActiGraph accelerometers J Appl Physiol 2008 105 4 1091 1097 10.1152/japplphysiol.90641.2008 18635874 PMC2576046

[B29] Chen KY Bassett DR Jr The technology of accelerometry-based activity monitors: current and future Med Sci Sports Exerc 2005 37 11 Suppl S490 S500 16294112 10.1249/01.mss.0000185571.49104.82

[B30] Cole TJ Bellizzi MC Flegal KM Dietz WH Establishing a standard definition for child overweight and obesity worldwide: international survey BMJ 2000 320 7244 1240 10.1136/bmj.320.7244.1240 10797032 PMC27365

[B31] Eslinger D Mota J Silva P Welk G Technical reliability assessment of the Actigraph GT1M accelerometer Meas Phys Educ Exerc Sci 2010 14 2 79 91 10.1080/10913671003715524

[B32] Santos-Lozano A Marín PJ Torres-Luque G Ruiz JR Lucía A Technical variability of the GT3X accelerometer Med Eng Phys 2012 34 6 787 790 10.1016/j.medengphy.2012.02.005 22417978

[B33] Bland JM Altman DG Statistical methods for assessing agreement between two methods of clinical measurement Lancet 1986 1 307 310 2868172

[B34] Ayen TG Montoye HJ Estimation of energy expenditure with a simulated three-dimensional accelerometer J Ambul Monit 1988 1 293 301

[B35] Bouten CV Van De Verboeket-Venne WP Westerterp KR Verduin M Janssen JD Daily physical activity assessment: comparison between movement registration and doubly labeled water J Appl Physiol 1996 81 1019 1026 8872675 10.1152/jappl.1996.81.2.1019

[B36] Coleman KJ Saelens BE Wiedrich-Smith MD Finn JD Epstein LH Relationships between Tritrac vectors, heart rate, and self-report in obese children Med Sci Sports and Exerc 1997 29 1535 1542 10.1097/00005768-199711000-00022 9372493

[B37] Eston RG Rowlands AV Ingledew DK Validity of heart rate, pedometry, and accelerometry for predicting the energy cost of children’s activities J Appl Physiol 1998 84 362 371 9451658 10.1152/jappl.1998.84.1.362

[B38] Trost RG Ward DS Moorhea SM Watson PD Riner W Burtke JR Validity of the CSA activity monitor in children Med Sci Sports and Exerc 1998 30 629 633 10.1097/00005768-199804000-00023 9565947

[B39] Kinnunen H Tanskanen M Kyröläinen H Westerterp KR Wrist-worn accelerometers in assessment of energy expenditure during intensive training Physiol Meas 2012 33 1841 10.1088/0967-3334/33/11/1841 23110981

[B40] Westerterp KR Physical activity assessment with accelerometers Int J Obes (Lond) 1999 23 S45 S49 10.1038/sj.ijo.0800883 10368002

[B41] Plasqui G Joosen AM Kester AD Goris AH Westerterp KR Measuring free-living energy expenditure and physical activity with triaxial accelerometry Obes Res 2005 13 1363 1369 10.1038/oby.2005.165 16129718

[B42] Howe CA Staudenmayer JW Freedson PS Accelerometer prediction of energy expenditure: vector magnitude versus vertical axis Med Sci Sports Exerc 2009 41 2199 2206 10.1249/MSS.0b013e3181aa3a0e 19915498

[B43] Welk GJ Blair SN Wood K Jones S Thompson RW A comparative evaluation of three accelerometry-based physical activity monitors Med Sci Sports Exerc 2000 32 489 497 10.1097/00005768-200009001-00008 10993419

[B44] Hänggi JM Philips LR Rowlands AV Validation of the GT3X Actigraph in children and comparison with the GT1M Actigraph J Sci Med Sport In press 10.1016/j.jsams.2012.05.012 22749938

[B45] Vanhelst J Zunquin G Theunynck D Mikulovic J Bui-Xuan G Beghin L Equivalence of accelerometer data for walking and running: treadmill versus on land J Sports Sci 2009 27 7 669 675 10.1080/02640410802680580 19424900

[B46] Rowlands A Stone M Eston R Influence of speed and step frequency during walking and running on motion sensor output Med Sci Sports and Exerc 2007 39 4 716 27.0 10.1249/mss.0b013e318031126c 17414811

